# Tuberculosis in Nueses and Ward Maids

**Published:** 1924-10

**Authors:** 


### TUBERCULOSIS IN NURSES AND WARD MAIDS.

A recent statistical investigation, conducted by Dr. Scheel, into the incidence of tuberculosis among nurses and ward maids in the public hospitals of Christiania in the period 1918–1923 has brought out some instructive facts. Of the 2,913 nurses and 1,411 ward maids employed during this period, 63 of the former and 23 of the latter developed some form of tuberculosis while in hospital service. About half these cases were pleurisies, and it was noteworthy that among 27 cases of pleurisy in nurses who had served from one to eight years there were as many as 15 occurring during the first year of service. Why is this first year so unfortunate in this respect ? Dr. Scheel points out that this first year is one of new and strenuous labour undertaken by girls who have just reached 20, and have come, in many cases, from the country, where they may not hitherto have been exposed to infection with tuberculosis. With a 9-hour work day (not counting night duty, which entails even longer hours) these probationers have a hard time of it bodily and mentally. Dr. Scheel's remedy for this state of affairs is both practical and simple. He suggests that every probationer shall be tested with Pirquet's tuberculin reaction on joining a hospital, and that when she gives a negative reaction, and thus shows herself to be virgin soil, she shall not for the first year of service be made to nurse tuberculous patients.

Dr. Scheel incidentally points out that while the raw probationer, untrained in heavy work, is made to toil nine hours a day or more, and ward maids are expected to work for as long, the hours of hospital porters are only 8¼. We do not grudge the porters their conditions of work, but it seems hard that the life of the probationer nurse should be so strenuous. Nursing veterans with constitutions of iron may speak with complacency of the " good old days," when the chaff was weeded out by a process inaccurately described as " the survival of the fittest." It is no exaggeration to say that in this country there must be thousands of potentially useful nurses who have been lost to the profession because the conditions of their first year of service were made too hard for them, and among them there must be many who have been broken by tuberculosis.

### Professional Classes Aid Council.

The Professional Classes Aid Council's annual report recently issued states that there is no lessening in the volume of distress with which the Council has to deal, and no change of policy has been found necessary. The number of applications dealt with during the year was 685, and 352 individuals were given financial help, while the expenditure on education was over £1,500. The year's expenditure exceeded income by £267.

